# System approaches to childhood obesity prevention: ground up experience of adaptation and real-world context

**DOI:** 10.1017/S1368980022002531

**Published:** 2023-04

**Authors:** Penny Fraser, Jillian M Whelan, Andrew D Brown, Steven E Allender, Colin Bell, Kristy A Bolton

**Affiliations:** 1 Global Centre for Preventive Health and Nutrition (GLOBE), Institute for Health Transformation, School of Health and Social Development, Deakin University, Geelong, VIC, Australia; 2 Global Centre for Preventive Health and Nutrition (GLOBE), Institute for Health Transformation, School of Medicine, Deakin University, 1 Gheringhap St, Geelong, VIC, Australia; 3 Institute for Physical Activity and Nutrition, School of Exercise and Nutrition Sciences, Deakin University, Geelong, VIC, Australia

**Keywords:** Childhood obesity prevention, Complexity, Intervention, System thinking, Process adaptation

## Abstract

**Objective:**

Childhood obesity prevention is critical to reducing the health and economic burden currently experienced by the Australian economy. System science has emerged as an approach to manage the complexity of childhood obesity and the ever-changing risk factors, resources and priorities of government and funders. Anecdotally, our experience suggests that inflexibility of traditional research methods and dense academic terminology created issues with those working in prevention practice. Therefore, this paper provides a refined description of research-specific terminology of scale-up, fidelity, adaptation and context, drawing from community-based system dynamics and our experience in designing, implementing and evaluating non-linear, community-led system approaches to childhood obesity prevention.

**Design:**

We acknowledge the importance of using a practice lens, rather than purely a research design lens, and provide a narrative on our experience and perspectives on scale-up, fidelity, context and adaptation through a practice lens.

**Setting:**

Communities.

**Participants:**

Practice-based researcher experience and perspectives.

**Results:**

Practice-based researchers highlighted the key finding that community should be placed at the centre of the intervention logic. This allowed communities to self-organise with regard to stakeholder involvement, capacity, boundary identification, and co-creation of actions implemented to address childhood obesity will ensure scale-up, fidelity, context and adaptation are embedded.

**Conclusions:**

We need to measure beyond primary anthropometric outcomes and focus on evaluating more about implementation, process and sustainability. We need to learn more from practitioners on the ground and use an implementation science lens to further understand how actions work. This is where solutions to sustained childhood obesity prevention will be found.

Obesity costs the Australian economy more than $8·6 billion per year and is a risk factor for multiple cancers and heart disease^(1)^. Prevention is a key; however, community-based prevention is often limited by complex and changing risk factors, insufficient resources and changing priorities of governments and funders.

System science has emerged as an approach to help health promoters manage this complexity and engage community stakeholders as partners in disease prevention. When partnerships where communities have real power in the design and implementation of local disease prevention work, we see healthy changes in behaviours, quality of life measures and childhood obesity prevalence^(2)^. We are yet to see examples of sustained long-term impact to date^(3)^; however, ideally community capacity is built so that skills remain when researchers leave^(4)^.

While system science provides an opportunity to build capacity, our experience working with eleven communities suggests that inflexibility of traditional research methods and dense academic terminology creates issues with those in prevention practice.

This ‘in practice’ article describes how we, as practice-based researchers, are endeavouring to be more flexible, use more inclusive language and strengthen community capacity. Firstly, the authorship group includes practitioners, health service-based researchers and university-based researchers. Secondly, within the article, we draw and adapt language and practice from community-based system dynamics (CBSD)^(2)^. CBSD was purposefully chosen due to the methodological alignment with core community principles such as community engagement, capacity building and empowerment of communities to understand and solve complex problems which emphasise where they have power and influence to act^(2)^. In CBSD, academic and community partners work together to understand the local systems and find potential places to intervene^(2)^. CBSD involves collectively mapping the drivers of the issue into a causal loop diagram, using a tool such as Systems Thinking in Community Knowledge Exchange^(5)^, and helps develop solutions to problems defined and directed by the community itself; these solutions are by definition contextually relevant to the community in which they are developed^(2)^. Finally, we acknowledge the need for scientific rigour but create room to reconceptualise terms such as scale-up, fidelity, context and adaptation through a practice lens. This paper aims to provide a more nuanced understanding of research implementation terminology and processes drawing from our experience in designing, implementing and evaluating non-linear, community-led approaches to childhood obesity prevention.

## Where are we now?

Swinburn *et al*. (2019) characterised the evolution of community-based prevention in three stages: package delivery, capacity building and system approaches^(3)^. A recent example of a third-stage system approach was The Whole of Systems Trial of Prevention Strategies for Childhood Obesity (WHO STOPS)^(5)^. WHO STOPS demonstrated a reduction in childhood overweight/obesity in the first 2 years and improvements in intervention children’s takeaway (fast food) consumption and health-related quality of life over 4 years^(5)^. WHO STOPS utilised CBSD to identify and prioritise obesity prevention actions^(6)^ and build community capacity by training community members in CBSD and employing an implementation specialist from the community who supported the community to prioritise and adapt actions based on relevance and feasibility for the community^(6)^. This approach was welcomed by members of the community as it creates momentum and engagement with other sectors not traditionally included in the work^(7)^.

## Conceptualising scale-up, fidelity, context and adaptation in light of CBSD

We recognise the need for empirical evidence of intervention effectiveness from tightly controlled, high-fidelity trials and for interventions to subsequently be tested at different scales and in diverse community contexts. CBSD presents a rigorous research design and process but deliberately seeks adaptive responses tailored to local context and so considering interventions as a single package replicated at any scale, with a focus on fidelity of delivery, requires different re-conceptualisation to allow for context and adaptation.

## Scale-up

Scaling up refers to ‘deliberate efforts to increase the impact of successfully tested health interventions so as to benefit more people and to foster policy and programme development on a lasting basis’^(8)^. Scale is intrinsically included in the design of CBSD as the process begins by setting the geographical, cultural and fiscal boundaries within which the community will work^(9)^. In this way, CBSD encourages participants to adapt to the scale as defined by boundaries such as the focus/target issue they want to address, the populations they want to reach, community representation required to tackle the issue and the geographical boundary to work within. System dynamics projects have addressed large-scale global issues such as climate change^(9)^ and small-scale issues such as helping an individual consider how to improve their own life^(2)^. CBSD does not rely solely on identifying specific actions to deliver at differing scales, but rather encourages participants to adapt, refine and evolve at the scale for which they have the power to act. Scaling up CBSD may be running workshops and capacity-building efforts repeatedly across communities, but it can also mean working with communities to consider influencing change at the state, national or international scale or working with leaders at different levels to carry out system change across levels. It is important that the community decides who is in the room and what actions are prioritised for implementation in line with community resources and local impact.

## Fidelity

Fidelity refers to the degree to which an intervention is implemented and delivered as planned^(10)^. The concept of fidelity and its definition varies greatly within the literature^(10)^. In our work, fidelity relates to fidelity of the CBSD process rather than fidelity to a pre-defined programme or list of actions. The community determines the issue they are addressing, self-organises around attendance at sequential specified workshop activities and decides which actions are most appropriate to implement. There is no guidance on sample size or demographic composition of the community participants in CBSD, beyond a call for representativeness of the population who is the target of the intervention.

Following Hawe^(11)^, we agree that the function and process of the intervention should be standardised; for example, in CBSD, workshop activities are standardised, through the use of evidence-based scripts^(12)^ from the literature and traditional public health tools, and metrics are adapted to assist in understanding community and system-level change. We also agree with Hawe’s^(11)^ suggestion that the components themselves should not be standardised – in CBSD, this would relate to community actions. The actions are co-created and driven by the community to solve their defined issue. Each community creates actions differently, drawing from different resources available, and contextually tailors them for implementation and evaluation.

## Context

Community context includes the dynamic factors which influence community life, that is, the local socioeconomic conditions, community history, community infrastructure (e.g. access to services, amenities, relationships within the community, social connections, networks), access to resources, social capital, macrosystem policies, ecology, capacity, culture and perceptions of community strengths and stigma^(13,14)^. In implementation science, context is acknowledged as important, but is framed as something to be managed to maintain fidelity. The standard approach to addressing context is randomisation to allow all other issues, other than the intervention components, to be balanced between intervention and comparison arms. Context often ends up being blamed for a loss of intervention effectiveness as context differs between pilot sites and scale-up sites^(15)^.

The community in CBSD is self-identifying and self-organising^(2)^. The context, including direct elements (geographical, demographic, economic, environmental) and indirect elements (political, relationships, history of success), is embedded throughout the CBSD approach. Community members are immersed within the context. Therefore, the direct context is considered by the community when discussing the variables that influence childhood obesity, the design of the causal loop diagram and the co-creation of feasible and tailored actions to implement in the community. The context defines the boundary the community works in and directs the process and issue being prioritised.

## Adaptation

Adaptation can be defined as modifying to meet the needs of the target population, local circumstances or new contexts^(16)^. Adaptation within CBSD occurs implicitly in two important ways. Firstly, our experience has shown that communities themselves collectively come to a consensus regarding the specific topic or question to focus on in workshops. This nuance of variation allows for each community to maximise their local success and ensure engagement and empowerment of community members involved while fidelity to process remains firm. Examples of variations include factors that influence healthy eating and physical activity in our community (in children) and factors that influence our local children in reaching their full potential

Secondly, adaption is built into the process from the beginning of CBSD approaches; in that, the causal loop diagram is not fixed but a snapshot in time that continues to change and evolve.

## Where to from here?

A deeper examination of intervention logic underpinning CBSD approaches will build stronger, more effective interventions^(17)^. This will involve moving beyond only measuring primary outcomes like zBMI and ensuring aspects such as the implementation process (why or why not were actions implemented, barriers/enablers to implementing action, how did community context/priorities/readiness/engagement/relationships impact implementation of actions) and process evaluation are considered. Additionally, sustainability, often considered within process evaluation, is important to measure. We also need to reconceptualise scale-up, fidelity, context and adaptation with a practitioner lens. The next generation of interventions will grow from scrutiny and understanding of real-world application of CBSD approaches mobilising community-owned and led action. We need to learn from practitioners on the ground and use an implementation science lens to further understand how actions work. What was most effective? What action/s were sustainable over time and what improvements for the future can be made? There are also learnings to be gained in how information is disseminated from CBSD more widely, so efforts and knowledge are more readily shared. Through an informed community, that understands the causes and interactions of their identified issue, they can generate action that is effective and sustainable. Looking beyond zBMI (or BMI), to more long-term sustainable measures, allows for a community to build momentum for complex issues. New tools to support communities to do this quickly, and with high utility, are also needed, such as Systems Thinking in Community Knowledge Exchange^(12)^, a tool to support communities to not only visualise their complex issue by building a causal loop diagram but also enable communities to track action over time and plan to capture outcomes and community indicators related to implementation of actions.

CBSD can help overcome the limitations of simple and linear programmatic responses to complex problems like childhood obesity as it creates co-designed local and context-adapted actions to allow for the non-linear relationships and unintended consequences, delays, and feedback system experience^(2)^. However, we need to be cautious because system interventions; similar to any type of intervention; are vulnerable to shortcuts, funding cycles and turnover within communities. For example, CBSD approaches can rely on reorienting existing resources to use more effectively, but if a community has limited resources or competing priorities, for example unexpected economic or environmental shocks^(5)^, they may not prioritise this work and therefore not sustain momentum.

## Conclusion

Community self-organisation and self-identification of boundaries, co-creation of actions to address the complex problem of childhood obesity to embed scale-up, fidelity, context and adaptation are key characteristics of CBSD approach. Here we argue for their importance but through a practice lens rather than research design lens. Solutions to sustained childhood obesity, in our view, will be found in community identification and adaption of actions, with fidelity of process and the scale and context of ‘the’ community placed at the centre of the entire logic.

## References

[1] Price Waterhouse Cooper (2015) Weighing the Cost of Obesity: A Case for Action. https://www.pwc.com.au/pdf/weighing-the-cost-of-obesity-final.pdf (accessed March 2021).

[2] Hovmand P (2014) Community Based System Dynamics. New York: Springer.

[3] Swinburn BA , Kraak VI , Allender S et al. (2019) The global syndemic of obesity, undernutrition, and climate change: the Lancet Commission report. Lancet 393, 791–846. 30700377 10.1016/S0140-6736(18)32822-8

[4] Millar L , Robertson N , Allender S et al. (2013) Increasing community capacity and decreasing prevalence of overweight and obesity in a community based intervention among Australian adolescents. Prev Med 56, 379–384. 23485797 10.1016/j.ypmed.2013.02.020

[5] Allender S , Crooks N , Bolton KA et al. (2021) Four-year behavioral, health-related quality of life, and BMI outcomes from a cluster randomized whole of systems trial of prevention strategies for childhood obesity. Obesity 29, 1022–1035. 33950583 10.1002/oby.23130 PMC8251751

[6] Allender S , Millar L , Hovmand P et al. (2016) Whole of systems trial of prevention strategies for childhood obesity: WHO STOPS childhood obesity. Int J Environ Res Public Health 13, 1143. 27854354 10.3390/ijerph13111143 PMC5129353

[7] Whelan J , Love P , Millar L et al. (2019) A rural community moves closer to sustainable obesity prevention – an exploration of community readiness pre and post a community-based participatory intervention. BMC Public Health 19, 1420. 31666042 10.1186/s12889-019-7644-x PMC6820900

[8] World Health Organization & Department of Reproductive Health and Research (2010) Nine Steps for Developing a Scaling-Up Strategy. Geneva: WHO Press.

[9] Meadows DH , Meadows DL & Randers J (1992) Beyond the Limits: Confronting Global Collapse, Envisioning a Sustainable Future. Vermont: Chelsea Green Publishing Co.

[10] Breitenstein SM , Gross D , Garvey CA et al. (2010) Implementation fidelity in community-based interventions. Res Nurs Health 33, 164–173. 20198637 10.1002/nur.20373 PMC3409469

[11] Hawe P , Shiell A & Riley T (2004) Complex interventions: how “out of control” can a randomised controlled trial be? BMJ 328, 1561–1563. 15217878 10.1136/bmj.328.7455.1561 PMC437159

[12] Hovmand PS , Andersen DF , Rouwette E et al. (2012) Group model-building ‘scripts’ as a collaborative planning tool. Syst Res Behav Sci 29, 179–193.

[13] South J , Bagnall AM , Trigwell J et al. (2020) Complexity and community context: learning from the evaluation design of a national community empowerment programme. Int J Environ Res Public Health 17, 91. 10.3390/ijerph17010091 PMC6981559 31877710

[14] Trickett EJ , Beehler S , Trimble JE et al. (2011) Advancing the science of community-level interventions. Am J Public Health 101, 1410–1419. 21680923 10.2105/AJPH.2010.300113 PMC3134512

[15] Ballard E , Farrell A & Long M (2020) Community-based system dynamics for mobilizing communities to advance school health. J School Health 90, 964–975. 33184879 10.1111/josh.12961 PMC7702041

[16] Solomon J , Card JJ & Malow RM (2006) Adapting efficacious interventions: advancing translational research in HIV prevention. Eval Health Prof 29, 162–194. 16645183 10.1177/0163278706287344

[17] Hawe P (2020) Scaling-Up Complex Interventions: Adaptation is Not a Threat to Fidelity. University of Sydney, Faculty of Medicine and Health. https://ses.library.usyd.edu.au/handle/2123/23109 (accessed October 2020).

